# Barriers to Implementing the Kidney Disease Outcomes Quality Initiative End-Stage Kidney Disease Life Plan Guideline

**DOI:** 10.1007/s11606-023-08290-5

**Published:** 2023-07-05

**Authors:** Michelle S. Keller, Christine Mavilian, Keaton L. Altom, Kevin F. Erickson, Laura M. Drudi, Karen Woo

**Affiliations:** 1 Division of General Internal Medicine, Department of Medicine, Cedars-Sinai Medical Center, Los Angeles, CA USA; 2 Department of Health Policy and Management, Fielding School of Public Health, UCLA, Los Angeles, CA USA; 3 Division of Vascular Surgery, Department of Surgery, David Geffen School of Medicine, UCLA, Los Angeles, CA USA; 4 Department of Surgery, Tripler Army Medical Center, Honolulu, HI USA; 5 Section of Nephrology, Department of Medicine, Baylor College of Medicine, Houston, TX USA; 6 Division of Vascular Surgery, Centre Hospitalier de L’Université de Montréal (CHUM), Montreal, QC Canada; 7 Innovation Hub, Centre de Recherche du CHUM, Montreal, QC Canada

**Keywords:** dialysis access, shared decision-making, life plan, end-stage kidney disease

## Abstract

**Objective:**

The updated 2019 National Kidney Foundation Kidney Disease Outcomes Quality Initiative vascular access guidelines recommend patient-centered, multi-disciplinary construction and regular update of an individualized end-stage kidney disease (ESKD) Life-Plan (LP) for each patient, a dramatic shift from previous recommendations and policy. The objective of this study was to examine barriers and facilitators to implementing the LP among key stakeholders.

**Methods:**

Semi-structured individual interviews were analyzed using inductive and deductive coding. Codes were mapped to relevant domains in the Consolidated Framework for Implementation Research (CFIR).

**Results:**

We interviewed 34 participants: 11 patients with end-stage kidney disease, 2 care partners, and 21 clinicians who care for patients with end-stage kidney disease. In both the clinician and the patient/care partner categories, saturation (where no new themes were identified) was reached at 8 participants. We identified significant barriers and facilitators to implementation of the ESKD LP across three CFIR domains: Innovation, Outer setting, and Inner setting. Regarding the Innovation domain, patients and care partners valued the concept of shared decision-making with their care team (CFIR construct: *innovation design*). However, both clinicians and patients had significant concerns about the complexity of decision-making around kidney substitutes and the ability of patients to digest the overwhelming amount of information needed to effectively participate in creating the LP (*innovation complexity*). Clinicians expressed concerns regarding the lack of existing evidence base which limits their ability to effectively counsel patients (*innovation evidence base*) and the implementation costs (*innovation cost).* Within the Outer Setting, both clinicians and patients were concerned about *performance measurement pressure* under the existing “Fistula First” policies and had concerns about reimbursement (*financing*). In the Inner Setting, clinicians and patients stressed the lack of *available resources* and *access to knowledge and information*.

**Conclusion:**

Given the complexity of decision-making around kidney substitutes and vascular access, our findings point to the need for implementation strategies, infrastructure development, and policy change to facilitate ESKD LP development.

**Supplementary Information:**

The online version contains supplementary material available at 10.1007/s11606-023-08290-5.

## INTRODUCTION

The management of chronic diseases is complex, affects the daily lives of people who are impacted by chronic disease, and requires attention to the individual person’s specific needs and preferences.^1^ In 2001, the Institute of Medicine deemed person-centered care to be one of the six pillars of quality health care.^2^ Person-centered care represents a shift from the traditional paradigm where the healthcare professional is the primary decision-maker towards a model that prioritizes individual wishes and requirements^3^. While person-centered care has been shown to result in greater satisfaction with care and patient well-being, implementation of person-centered care continues to face multiple barriers, including the challenge of shifting traditional healthcare practices and structures^3^.

End-stage kidney disease (ESKD) is a chronic disease that affects more than 780,000 Americans.^4^ In the USA, hemodialysis is the most common substitute kidney, necessitating vascular access.^4^ The 2006 National Kidney Foundation Dialysis Outcomes Quality Initiative (NKF K-DOQI) Clinical Practice Guideline for Vascular Access,^5^ in conjunction with “Fistula First,”^6^ created an environment in which kidney care professionals were all encouraged to recommend arteriovenous fistula as the ideal hemodialysis vascular access for a given patient, regardless of the patient’s individual characteristics and preferences. However, with newer data that indicates that fistula outcomes are not necessarily superior in all patient subgroups, the 2019 update of the K-DOQI Guidelines advocates for a substantial shift to a more patient-centered approach.^7^

To promote shared decision-making and shift towards patient-centered care, the 2019 K-DOQI guidelines emphasize the “ESKD Life Plan,” a strategy for creating a plan for all substitute kidney methods and anticipated vascular access procedures for an individual patient, for the remainder of their hemodialysis-dependent life.^7^ Substitute kidney methods include peritoneal dialysis, hemodialysis (both in-center and home), transplant, and conservative care. Vascular access procedures include central venous catheters and arteriovenous fistulas and grafts. The Life Plan (LP) aims to maximize a patient’s options for kidney substitution and vascular access over the patient’s lifetime by planning ahead and considering contingencies. The LP is to be developed by the multidisciplinary clinician team (primary care provider, nephrologist, surgeon, interventionalist (including radiologists, nephrologists, cardiologists, and surgeons)) in conjunction with the patient. The LP represents a dramatic shift in guidelines from the “one-size-fits-all” approach of “Fistula First” in which patients were universally advised that the ideal vascular access was arteriovenous fistula. Implementing the LP may require considerable infrastructure and face significant barriers. However, little data exists regarding facility of LP implementation. Thus, the objective of this study was to determine the barriers and facilitators to LP implementation from the perspective of relevant patients and clinicians.

## METHODS

### Study Design

We used qualitative methods with grounded theory methodological orientation. The study was approved by the UCLA Institutional Review Board (IRB #20–001481). Interviews were conducted by PhD-trained interviewers (KW, MSK) using video teleconferencing (Zoom, Zoom Video Communications, San Jose, CA) or telephone and recorded using Tape-A-Call Pro (Teltech, New York, NY). A professional transcription company (Western Consultant Services) edited the Zoom-generated transcripts for accuracy and transcribed verbatim the recorded telephone calls.

### Patient Recruitment and Semi-structured Interview Guide

We recruited patients through the American Association of Kidney Patients, social media (e.g., Twitter), and the UCLA vascular surgery practice using purposeful and snowball sampling. Inclusion criteria were as follows: diagnosis of advanced kidney disease (including pre-dialysis, dialysis-dependent, or with a kidney transplant) and age ≥ 18. Exclusion criteria were as follows: not English- or Spanish-speaking; inability to understand the consent process and/or give consent; or currently institutionalized. We aimed to purposefully sample patient participants of heterogeneous age, sex, race/ethnicity, marital status, education level, employment, co-morbidities, experience with hemodialysis, and duration of kidney failure. We snowball sampled by asking recruited patients to recommend other patients. When a patient participant had a care partner that was highly involved in the patient’s care, available during the interview, and willing to be interviewed, the care partner was interviewed with the patient.

The patient semi-structured interview guide (SSIG) items were based on a previously used SSIG for a project focused on vascular access decision-making ^3^and refined to address preferences and attitudes about educational materials to support the vascular access decision-making process, engagement in constructing the ESKD LP, and partnering with clinicians and individuals involved in the decision-making process (Appendix 1). When patient participants were not familiar with the LP, the concept was explained by the interviewer and the LP form was shown to the participant. Throughout the course of interviewing and concurrent analysis, the SSIG was refined to further explore codes that investigators thought could contribute to potential themes.

### Clinician Recruitment and Semi-structured Interview Guide

Similarly, we recruited clinicians through the investigators’ professional networks and social media using purposeful and snowball sampling. We aimed to purposefully sample clinicians from different specialties, practice settings, geographic diversity, gender, and years in practice. US clinicians were eligible if they were currently caring for patients with kidney disease. We aimed to recruit four pre-planned groups of individual clinicians specified by the LP (primary care, interventional radiology, nephrology, surgery) and included other clinicians (e.g., nurses and coordinators) as we pursued certain lines of inquiry.

Clinician SSIG items focused on preferences and attitudes about the needed LP’s multidisciplinary execution, patient involvement, and clinical management of vascular access. We based the clinician SSIG on published barriers/facilitators of multidisciplinary teams, and the investigators’ extensive clinical experience in interacting with the specialty clinicians^8^ (Appendix 2). As above, the SSIG was refined throughout the interview and analysis process. Similarly, when clinician participants were not familiar with the LP, the interviewer explained the LP.

### Coding and Analysis

We analyzed patient and clinician interviews separately using inductive and deductive coding and Dedoose v9.0.54, a platform for organizing and analyzing research data (SocioCultural Research Consultants, LLC, Manhattan Beach, CA).^9^ Following Charmaz’s methodology, we used Dedoose to manually code and analyze transcripts as interviews were conducted.^9–11^ We created initial codes consisting of a short phrase generated by the investigator directly from the data with line-by-line coding.^9–11^ Codes were process codes using gerunds (“-ing” words) to describe participant action in the data.^12^ Initially, each interview transcript was independently coded by two investigators (KW, MSK). Investigators reviewed the double-coded transcripts and discussed coding discrepancies during bi-weekly investigator meetings until consensus.

When 6 interviews were coded in each participant category, the investigators observed, through constant comparison, repeated concepts in the data. Two investigators (KW, MSK) independently grouped the initial codes into focused codes (constructs that succinctly capture important pattern in the data in relation to the research question and are more conceptual in nature.)^9–11^ The larger investigator group reached consensus on the final list of focused codes. We applied the focused codes to the remaining transcripts, with KW and MSK checking to ensure consistency between coders. This first part of the coding process was inductive.

We then used deductive methods and mapped focused codes specific to LP implementation to relevant domains in the 2009 Consolidated Framework for Implementation Research (CFIR), then subsequently updated to the 2022 CFIR.^13,14^ The CFIR is a comprehensive framework that provides a structure for describing factors critical to the implementation of an innovation. The domains include (1) Innovation, which describes the degree to which the innovation is cost-effective, evidence-based, easy or complex to implement, and advantageous to implement; (2) Outer Setting, or the larger context in which the intervention is being implemented, which may include local, regional, and national contextual factors; (3) the Inner Setting, or the context of the organization in which the innovation is being implemented; (4) the Individuals, or the roles and characteristics of the individuals implementing the intervention; (5) and the Implementation Process, or the activities and strategies used to implement the innovation. Not all domains or constructs with a domain may be relevant to particular innovation implementation. We used constant comparison to analyze the data with techniques including examining negative cases (e.g., participants who had perceptions that contrasted significantly from others); contrasting perceptions and views from participants in different settings or specialties; contrasting patient and clinician perspectives; and examining emotions, language, and use of metaphors/similes. ^10,11,13,15,16^ (Further details regarding consolidated criteria for reporting qualitative (COREQ) studies available in Appendix 3).^17^

## RESULTS

We interviewed 21 clinicians, 11 patients and 2 care partners between 4/2021 and 6/2022 (Table 1). Interviews lasted 40–60 min. During analysis, we looked for any indication that clinician participants from a particular specialty expressed unique experiences or concerns and whether there were discrepancies among participants within a specialty that would require further exploration and found none. Very few clinicians and no patient participants had pre-existing knowledge of the LP. We identified barriers and facilitators across three 2022 CFIR domains: Innovation, Outer Setting, and Inner Setting. Within each domain, we categorized barriers/facilitators into CFIR constructs (italicized below), although we note that some findings fit into multiple CFIR constructs.

**Table 1:** Participant demographics

	Clinicians *n* = 21 (%)	Patients/care partners *n* = 13 (%)
Mean age (range)	48 (37–63)	50 (26–77)
Male	10 (47.6)	4 (30.8)
Race		
White Black Asian Multiple	13 (61.9) 2 (9.5) 4 (19) 2 (9.5)	7 (53.8) 2 (15.4) 2 (15.4) 1 (7.7)
Hispanic	1 (4.8)	1 (7.7)
State of residence		
CA	4 (19)	7 (53.8)
FL	2 (9.5)	1 (7.7)
GA	2 (9.5)	
ID		1 (7.7)
MI	2 (9.5)	
NC	1 (4.8)	
OH		1 (7.7)
PA	4 (19)	
TN	1 (4.8)	
TX	2 (9.5)	2 (15.4)
VA		1 (7.7)
WA	1 (4.8)	
WI	2 (9.5)	
Specialty		NA
Primary care^1^ Interventional radiology Nephrology Surgery Other^2^	3 (14.3) 3 (14.3) 4 (19) 7 (33.3) 4 (19)	
Practice setting		NA
Academic Private practice Private practice with academic affiliation Hospital-employed Veterans Affairs	8 (38) 7 (33.3) 3 (14.3) 1 (4.8) 2 (9.5)	
Experience with substitute kidney	NA	
Hemodialysis Peritoneal dialysis Transplant		10 (76.9) 6 (46.2) 7 (53.8)
Education	NA	
Some college College Graduate		6 (46.2) 4 (30.8) 3 (23.1)
Employment	NA	
Full time Part time Retired Not working		5 (38.5) 1 (7.7) 3 (23.1) 4 (30.8)
Co-morbidities	NA	
Asthma Cancer Diabetes Heart disease Hypertension Lung disease Stroke Other^3^		3 (23.1) 0 3 (23.1) 4 (30.8) 4 (30.8) 1 (7.7) 2 (15.4) 5 (38.5)
Current substitute kidney	NA	
Hemodialysis Peritoneal dialysis Transplant Caregiver/NA		4 (30.8) 1 (7.7) 6 (46.2) 2 (15.4)

^1^Trained in internal medicine followed by geriatrics fellowship

^2^Advanced Practice Provider, Physician Assistant, Vascular Access Coordinator, Nephrology Nurse

^3^Pulmonary hypertension, obstructive sleep apnea, spinal injury, lupus, seizure disorder

### Domain: Innovation

The “Innovation” domain refers to the intervention being implemented. Clinicians cited concerns about the evidence basis for LP construction, relevant to the CFIR construct *innovation evidence base*. Clinicians of various specialties noted lack of evidence to make vascular access decisions based on comorbidities, age, and other clinical and patient characteristics. One nephrologist expressed a desire for a clinical calculator or decision-aid that would assist with the vascular access decision-making process. Further, nephrologists noted the challenges of determining both the timing of initiation of substitute kidney and vascular access. Nephrologists repeatedly referred to the variable rate of ESKD progression, lack of reliable prediction tools in this area, and reluctance to initiate conversations too early, which could cause undue emotional distress for patients (Representative quotes in Tables 2, 3 and 4).

**Table 2:** Representative participant quotations relevant to CFIR domain: innovation domain

*CFIR sub-domains*	Clinicians	Patients
*Innovation evidence base* validity of the evidence supporting the ESKD Life Plan and whether the intervention will have the desired outcomes	I think the problem with all this is that we have no clue who’s going to succeed on dialysis and who’s not we have no clue who’s gonna have a fistula mature or not, and we have no clue who’s gonna die in the first two years…I’m just not convinced that this is the right way. We need more research, we need to understand the physiology, we need to do a lot more of that kind of work. *Clinician 2 (nephrologist)* If you're in the hospital endlessly for problem, after problem, after problem – which does happen, and you're thinking like what are we doing here? … I really think the ESKD Life Plan gives anybody that is really interested in taking care of their dialysis patient a foot to stand on, in my opinion, I think this is what we should do because patient wants this, daughter wants this, I think it's the best we're going to proceed on and yeah you'd like a fistula for your center but a graft is better, and so this is why we're doing it. *Clinician 9 (surgeon)*	None identified
*Innovation complexity:* Perceived difficulty of implementing the ESKD Life Plan, for example, the degree of disruptiveness, scope, duration, and number of steps to implement	It takes a lot of time and sympathy to create a very, very good rapport with a patient so that they will believe that what you’re trying to tell them is in their best interest…I don’t even know if the vascular surgeons could fit in their schedule a clinic for something like this, to be honest with you. *Clinician 4* (*nephrologist)* I think to get to have these sort of really formal sit-down meetings, that is maybe a hard thing to accomplish. *Clinician 6 (nephrologist)* I just again worry that patients are not always as engaged or understanding of it or become very overwhelmed by all their other problems that they may not be able to provide the information. *Clinician 7 (surgeon)* I can tell you that it is difficult enough just to get them focused on what is happening with them now. *Clinician 14* (*nurse)* I think that’s going to be kind of a big leap. I would say on average, the average patient that I see is probably not ready for that type of an advanced planning a long-range commitment type of plan for their kidney care. I mean it’s great for the well informed, motivated, educated patient – but I don’t think that is the typical patient and I know that sounds awfully cynical but I think it’s just reality. *Clinician 15 (surgeon)*	I feel like that would be overwhelming in a way. When you're going through it, you want the decision that's right in front of you. That is all you are worried about. You are not thinking about kind of the second order. *Patient 2* I mean anything related with surgeries or kidney related stuff can be stressful, overwhelming, causing anxiety. Especially when you're talking about surgeries. But fistulas, grafts, all those type of things can be stressful. *Patient 6* I’m sure they would feel overwhelmed. I’m sure they would feel…you know, a lot of these patients that I even talk to, are surprised, even though, like, “Am I going to be on dialysis for the rest of my life?” A lot of patients don't even know that. Especially the ones who are not eligible for transplant. *Patient 8* Our attitude has always been, we're just going to take it as it comes. *Patient* 9
*Innovation design:* Perceived excellence in how the intervention is bundled, presented, and assembled	What I think it does, though, is if it is going to be implemented correctly, it does prepare the staff and the patient for that access. And if that access fails, what is your follow up plan? So, it goes through, “alright, well if this doesn’t work, if I start with PD once my follow up when the PD catheter doesn’t work.” So, it gets you thinking about the future. *Clinician 14 (nurse)*	I think it would be helpful to have kind of that initial discussion at the beginning say, “Hey, if your numbers got to this, we're going to have this game plan and we've got kind of these things to figure out. But we will talk about it then.” *Patient 2* Well, I think that's going to be very helpful for patients. First of all, giving them the full education of what it means to have kidney disease. *Patient 4* People don't refer it to as a modality. They refer it as a treatment.- *patient 5* The information needs to be able to be personalized. More individualized to have its greatest impact. *Patient 9*
*Innovation cost*	There’s just no financial case for this. If you really think about the financial structure that you need to implement this, like how much money it would cost and how many patients they need to put through this, and not only that, but do we actually know that these actually influence decisions at the margin? *Clinician 2 (surgeon)* I don't know who is going to pay to have a team meeting for every patient. It sounds wonderful, but I don't see how it could possibly happen. *Clinician 5 (surgeon)*	None identified

**Table 3:** Representative participant quotations relevant to CFIR Domain: outer setting

*CFIR sub-domains*	Clinicians	Patients
*Local attitudes and conditions:* The extent to which the needs of individuals with end-stage renal disease are acknowledged by organizations. For example, are patient choices provided, patient barriers addressed, and patients have a high satisfaction with service	But I think as a medical community, we don't take a lot of that into consideration anymore. We don't really listen to the social situations as much as maybe we used to. Because we kind of, I don't know, this is all speculation in my mind. But it's possible that we feel by doing that, we’re intervening too much of our own preconceived notions about their situations and what they want and their ideals. *Clinician 20 (interventional radiologist)*	I actually had to do my own research… That's when I found out that home hemo dialysis was an option… And I came back to my units and stuff, and they are like, “Well, we've heard about it, but it's not been offered.” And I just kept thinking to myself, “Why not?” *Patient 5* But the thing is, ultimately, it just wasn't offered to me now. And I know that there's peritoneal dialysis, as well… But I wish it would have been because, like I said, I would have probably opted to do that right away, because of the liberty that it gave me as a patient. *Patient 6* Oh, you know what my vascular surgeon said? He said we're putting it in the right arm… You don't have a choice…They were just push, and push, and push it all the time for these things to be done, but they never stop to explain or have an explanation to me about what a fistula is. *Patient 7* So, she actually never mentioned PD to me at all… *Patient 8*
*Policies and laws:* Perception of how external policies and incentives will help or inhibit implementation of the ESKD Life Plan	One, I think the revision of the guidelines away from the “fistula first” I think is right on, and a huge thing. I think too much emphasis, I actually think it was not setting up patients for success when there was so much emphasis for the nephrologist in the dialysis centers to have a certain percentage of fistulas and stuff like that. And so, really patients who have been subjected to that push for fistulas with clearly inferior information, but I do think that idea of adopting a more personalized approach is very important, and was good. *Clinician 7 (surgeon)* I am in private practice so any kind of reimbursement I think would ring ears for some people, where if you were to say, “Okay I filled this, I did go through these steps”. I think that would go some way and in terms of—the other parts it's tough it's hard to coordinate with the surgeon on some of these things. But again, if there was something tied to that, whatever it might be conversation with the surgeon, and I documented this. *Clinician 10 (nephrologist)* It could even be one of those quality measures that for MIPS or whatever, if you had as one of those that if the patient needs a next access, what would that be? Just making somebody actually step back and think, “If I had to put in a different catheter tomorrow, where would I put it?” *Clinician 16 (interventional radiologist)*	None identified

**Table 4:** Representative participant quotations relevant to CFIR domain: inner setting

*CFIR sub-domains*	Clinicians	Patients
*Structural characteristics:* Organizational structures for collaboration between providers in healthcare for patients with ESKD	“First of all, academic centers allocate lots of money to things that are not profitable, so that’s one thing, and then, the second is they have huge patient panels, so they can justify large investments that pay off. That’s just not realistic. Your average nephrology private practice is small.” *Clinician 2 (surgeon)* [Private practice clinicians] are under time pressure. Meaning financial pressure to get these patients, if you're talking about outpatients, to get their outpatients into you and back to dialysis as quickly as possible. *Clinician 16 (radiologist)*	None identified
*Relational connections:* The presence and quality of formal and informal communication and networks between providers serving patients with ESKD	I think it is a lot more doable in probably a smaller community setting and in a smaller practice setting. Just because you have less players to get involved. It becomes a bit more complex when you are talking several surgeons, several IR’s, you know multiple, tens of nephrologists. *Clinician 17 (surgeon)*	None identified
*Tension for change:* Perceived need for change regarding current ESKD care planning	Some older patients don’t even want to be bothered with surgeries and having to have needles in their arms and all of that. So, their values definitely matter. *Clinician 4 (nephrologist)* I mean, the other big message, which I think is pretty new and a positive thing, is that you shouldn't just think about this first access. You should sort of think about what happens if that one fails. *Clinician 6 (nephrologist)* It's a lot about what their kind of wishes are for level of kind of, you know, how aggressive do they want their care to be, or what do they want. *Clinician 6 (nephrologist)* It is that next access that, in my experience, often gets neglected and people get tunnel vision. *Clinician 16 (interventional radiologist)*	So, I think most patients want it. I really do. I think most patients are scared… They don't know that there's modalities that they have better chances with than other modalities. And they want to learn…. I believe there's no education for patients and they're starving for it. *Patient 4* Well, I think that's going to be very helpful for patients. First of all, giving them the full education of what it means to have kidney disease. What it is going to look like for them dealing with this. *Patient 8*
*Compatibility:* The fit between the ESKD Life Plan intervention and the current structures and workflows	It’s something that more or less we’ve been doing… It’s talking with a patient about options, addressing those in time. *Clinician 4 (nephrologist)* …If anyone feels invested in a decision, then they are more likely to support and advocate for the plan or the outcome that you’ve decided. *Clinician 16 (interventional radiologist)*	And I think starting small and just saying, “Hey, we're going to do,” like you said, “A yearly visit. Yearly check in to see how you're doing, if anything has changed. How you're feeling about things. Do you have a preference on which direction you want to go, because what you said last year may be different from this year. *Patient 8*
*Available resources:* resources available for local implementation	And you know, I think certainly, I don’t know how, on a lot of EMRs I’m sure there are places that document can live and be able to be updated or reviewed. But… patients may need to carry that with them to their appointments to say this is where we’re at. *Clinician 7 (surgeon)*	A lot of people, sometimes a social worker, at the dialysis center will spend five minutes with you. That's not enough time to go over things. *Patient 5*
*Access to knowledge and information:* Ease of access to information, knowledge and support about how to implement the ESKD Life Plan	It is just the constant follow up and it is really dependent upon, right now, upon humans to make sure that the information is input. If something happens with the access, that the information is there. And sometimes, that will fall by the wayside. So that would be the downfall. *Clinician 14 (nurse)*	

Both clinicians and patients had significant concerns about the complexity of decision-making around substitute kidney and patients’ ability to digest the overwhelming amount of information and clinical terms needed to effectively participate in LP creation (*innovation complexity*). Clinicians noted that patients were often shocked and knew little about kidney failure when they were diagnosed, making advanced planning difficult. Beyond the initial diagnosis, the decision-making associated with setbacks was emotionally fraught. Moreover, clinicians perceived that often patients were reluctant to engage in complex shared decision-making and preferred to be told what to do in difficult situations.

Clinicians identified a variety of factors related to the *innovation complexity* of logistics surrounding LP implementation, including how the LP would be updated, who would be responsible for keeping it updated, how it would be shared across healthcare systems with different electronic health records (EHR), and the ability to assemble a multi-disciplinary team to discuss the LP (*available resources*). Clinicians cited language and cultural barriers as adding to the complexity of LP implementation, and that establishing rapport takes longer when these barriers exist.

Patients voiced similar concerns about the *innovation complexity*. When initially facing kidney substitution, patients recalled feeling “shocked,” “scared,” and so overwhelmed with that it was difficult to understand new information. While patients described slowly absorbing information over time, they described challenges dealing with setbacks and repeatedly having to make major decisions. The concept of planning ahead for major health decisions seemed daunting. Patients and care partners described a preference to manage only one decision at a time—the most imminent and necessary decision. Patients stressed that creating the LP might require repeated conversations over a long period of time, and many patients are likely not ready to have these difficult conversations, particularly when first diagnosed with kidney failure. However, a small minority of patients believed preparing patients for the future could possibly jolt patients into taking action. Patients’ beliefs about the complexity of the intervention also overlaps with the Outer Setting concept *local attitudes*, or the sociocultural values and beliefs that support or do not support implementation.

Clinicians and patients praised the relatively simple nature of the LP, relevant to *innovation design*. Clinicians embraced planning for subsequent accesses and documenting the plan to record patient preferences and facilitate communication between clinicians, particularly between institutions. Clinicians also supported detailing the patient’s clinical team members in the LP, enabling appropriate clinician communication.

Patients and care partners liked the personalized nature of the LP, contrasting it to existing brochures/pamphlets, but identified several areas where *innovation design* could be improved. Patients noted that some words in the current LP such as “modality” would need to be translated to more patient-friendly language. Others noted that the LP should be in a large text for patients with vision issues. One patient expressed concern that the linear structure of the existing ESKD LP suggests that patients could not go back to a previous decision or approach and suggested making the LP diagram a circle.

Clinicians, particularly those in private practice, worried about the *innovation cost* of LP implementation, without a reimbursement mechanism for the required effort, a concept which also falls into the Outer Setting CFIR construct of *financing*. One of the LP tenets is multi-disciplinary shared decision-making. This struck clinicians as extremely complex and potentially very costly to implement. In some academic practices, multi-disciplinary meetings are an existing element of the culture and are considered a routine component of employment. However, this is rare in private practice.

### Domain: Outer Setting

The “Outer Setting” of the intervention refers to the larger context in which the intervention is being implemented. This may include the local, regional, and national context. Relevant to *Iocal attitudes*, overwhelmingly, patients felt as though they were underestimated by the healthcare system, their preferences not valued, and they were largely uninformed about potential setbacks, such as kidney transplant failures, fistula failures, and peritonitis. Patients described not being offered modality choices, such as home-hemodialysis and peritoneal dialysis, and learning about these options months or years later from other patients, internet searches, or patient advocacy associations. Several non-White patients also noted that non-White patients were less likely to be offered these options; they perceived that clinicians made assumptions about their lack of ability to implement these modalities. Patients questioned whether financial incentives from dialysis facilities were responsible for the lack of choices and suspected that dialysis facilities profited more with in-center hemodialysis (*local conditions*).

Pertinent to *policies and laws* and *performance measurement pressure*, clinicians noted that current Centers for Medicare and Medicaid Services (CMS) ESKD Quality Incentive Program (QIP) policies^18^ are likely to impede clinicians’ autonomy to implement personalized patient care. Relatedly, patients reported being pressured by facility staff to transition from a catheter to a fistula. While the majority of clinician participants praised the move away from the “fistula-first” approach and towards a more personalized decision-making approach, a select few still strongly believed in “fistula-first” and reported that they did not offer patients many choices. Clinicians noted that there were currently no policies that would offer reimbursement or incentive programs which would facilitate implementation of the LP (*financing*). While several noted that reimbursement policies aligning with the LP would improve adoption, others noted that the time and effort would not be worth a small reimbursement.

#### Domain: Inner Setting

The “Inner Setting” refers to the setting where the implementation of the intervention is taking place. In this case, we used the term “Inner Setting” to describe the practice settings in which the ESKD LP would be implemented. Clinician participants described a variety of practice settings, including academic, private, hospital-employed, and Veterans Affairs facilities that reflected varied *structural characteristics* and *relational connections*, which could influence LP implementation. Clinicians practicing in larger organizations such as academic medical centers with nephrology clinics, interventional radiology, a dialysis facility, and vascular access surgeons within the same institution usually have a shared EHR. Clinicians noted this structure could facilitate implementation through communication via case conferences, sharing the LP across the EHR, and shared organizational LP rollout. Larger institutions may also have more resources to implement new programs. However, several clinicians noted that in their experience, large academic centers with many types of specialists and sub-specialists resulted in less continuity of care per provider and looser networks between clinicians, which was detrimental for patients.

Private practice community clinicians confirmed these sentiments, describing how their reliance on referrals fostered close working relationships with other clinician groups, which facilitated communication and strengthened ties between clinicians. However, challenges in *structural characteristics* in the private practice setting included significant pressures to see many patients and fewer resources to implement new programs including the LP.

Regarding implementation climate, patients and clinicians described significant *tension for change*. Both compared the relative advantage of the LP to the status quo, where little advanced planning occurs and decisions are made impromptu. Numerous clinicians had experienced cases where emergency-based decision-making led to poor patient outcomes. Patients described receiving little information from clinicians about kidney disease and their treatment options. Patients who worked with other patients, either in advocacy or patient education roles, emphasized the critical need to educate patients on different options, future decisions, and potential setbacks.

Clinicians were divided on the *compatibility* of the LP with their current practice. Some strongly believed in aligning decisions with patient values and reported already integrating shared decision-making into their practice. These clinicians already felt that it was critical to always consider the next access. Others questioned whether the effort and coordination required to create the LP would fit into current workflows. Some believed patients desire a more prescriptive approach over shared decision-making. Moreover, several clinicians felt that anatomy and other patient-specific clinical factors were major drivers in access decision-making, making it difficult to give patients choice. Patients embraced the opportunity to discuss their values with their care team and welcomed regular discussions about current values and preferences.

Clinicians and patients stressed the lack of *available resources* and *access to knowledge and information*. Limits on clinician time and availability severely restricts access to necessary time to implement the LP, including time with patients, documenting, and communicating with the other multi-disciplinary team clinicians. Patients repeatedly conveyed that they already experienced rushed visits with clinicians of all types and specialties. Numerous clinicians reported that their health care organizations already had limited numbers of staff, who were stretched to the limit. Further increasing the challenge would be the amount of training that would be needed to train all involved staff on the LP. Finally, clinicians noted that there are no existing resources or infrastructure to support the required constant LP updates at all of a patient’s healthcare delivery sites.

## DISCUSSION

This study elucidated barriers and facilitators to LP implementation by applying CFIR to patient and clinician perspectives. Patients and clinicians shared the view that the LP addressed important issues of lack of planning that often occurs with vascular access, lack of information that patients receive, and need to individualize care planning for patients with ESKD.

Clinicians raised significant concerns about lack of reliable evidence to inform timing of kidney substitute initiation and vascular access decision-making. One of the lead authors of the 2019 KDOQI guidelines has indicated that the currently quality of the evidence relevant to the access surgeon is limited.^19^ Similarly, despite numerous efforts to create prediction models for the timing of initiation of chronic kidney substitution, this task remains a significant challenge for clinicians to accomplish with any accuracy.^20^ Further, individualizing recommendations is difficult when little reliable evidence exists regarding variation in outcomes by specific patient characteristics. As several clinicians noted, age and frailty are often a primary consideration in making vascular access recommendations given frailty is associated with adverse treatment outcomes for ESKD with respect to kidney transplant^21^ and dialysis^22^ and is associated with increased risk of mortality.^23^ However, little data exists as to the association of frailty and vascular access outcomes.^24^

Both clinician and patient participants expressed concern about the ability of patients to comprehend and digest information required to adequately participate in the shared decision-making process around modality and vascular access planning. Other authors have demonstrated that pre-operatively, as little as 14% of patients are adequately “informed” about dialysis vascular access creation.^25^ Our previous qualitative work also found that patients lacked clear understanding about the types of access options and potential downstream consequences.^26^

Further contributing to the challenge of successful shared decision-making is the intense phenomenon of emotional overwhelm emphasized by both clinician and patient participants. “Emotional overwhelm” relates to “the emotional burden of the illness experience and consequent cognitive overload” and is distinct from “information overload,” which refers to “complexity, uncertainty, or volume of information involved in the decision.”^27^ Feeling both is common in patient experiences where the diagnosis and/or treatment may be life-changing, such as cancer or diabetes.^28,29^ Anxiety and other strong negative emotions can impair decision-making and increase passivity.^30,31^ Clinicians may need to address these emotions before proceeding with the decision-making process to mitigate lack of patient comprehension and engagement.

In our previous work, we found that as patients gain more experience with kidney substitution and with vascular access, their concerns and preferences shift towards issues specific to their individual experiences^26^. In an effort to provide a surrogate for experience to patients who are earlier in the kidney substitution journey, education and exposure to other patients’ experiences may assist with engaging patients in LP development. Similarly, it is critical that the LP be revised on a regular basis as a patient’s preferences and needs will likely change as they progress through the kidney substitution journey.

The commonly experienced emotional overwhelm in the ESKD population further raises the important issue of adequate emotional and psychological support. Dialysis-dependent patients report feeling bound to dialysis, feeling underrecognized and ignored with respect to mental health support, worrying about an uncertain future, developing self-reliance, and responding to a major lifestyle overhaul.^32^ Roughly 50% of patients with ESKD rely on dialysis experience symptoms of depression and anxiety.^33^ Approximately 27% of US adults with chronic kidney disease (CKD) have mental illness, and 7.1% have serious mental illness.^34^ Despite evidence demonstrating the effectiveness of psychological treatments such as cognitive behavioral therapy, exercise and relaxation techniques in treating depression in dialysis-dependent patients,^35^ few ESKD patients with severe mental health symptoms receive behavioral health treatment.^36^ An important component of LP implementation will be strategies to ensure patients with CKD/ESKD who need mental health services get the necessary care. Significant economic incentive and policy barriers exist to LP implementation. As was oft-noted by clinicians in our sample, the CMS “Fistula First-Catheter Last” initiative and ESKD QIP remain unchanged.^37^ Both clinician and patient participants noted a strong push from dialysis facilities to promote fistulas, reflecting the current policy incentives. Moreover, clinician participants noted the lack of reimbursement for implementing the LP and no healthcare institution or practice can remain functional without a positive financial balance. As long as these financial incentives and penalties exist, without adequate financial reimbursement for implementing the LP, there will be a conflict between guidelines and practice. A policy overhaul that prioritizes patient-centric care is critically needed to ensure adequate guideline implementation.

Importantly, our study identified a significant lack of knowledge of the LP among patients and clinicians. As participants emphasized, LP implementation will require significant clinician and staff training and processes for storing and sharing the document. Similar challenges were encountered in implementation of Physician Orders for Life Sustaining Treatment (POLST) forms, leading to the development of POLST registries which could be integrated into health care systems’ EHRs.^38,39^ A similar approach may be useful for the LP and will require state level or ideally, national efforts to realize.

A major strength of our study was the wide range of clinician specialties, locations, and practice settings. Additionally, we included patient participant perspectives which is often excluded from implementation studies. By including a broad range of clinician specialties and settings across the USA and patient perspectives, we have broadened the transferability of the study, an important concept in qualitative research that describes how the study’s findings could be applicable to other contexts, times, and populations. Limitations include the lack of interviews with operational managers, institutional executives, and payers who will need to play a significant role in the successful implementation of the LP. All interviews were conducted in English; while we had interviewers available who could have conducted the interview in Spanish, we did not recruit any participants who spoke Spanish exclusively. Future work will include efforts to involve non-English speakers.

## CONCLUSION

Patients and clinicians in our study support a tension for change from the status quo, but few large-scale organizational resources or efforts currently exist to implement the ESKD LP. From the clinician perspective, lack of effective, early planning puts patients at increased risk for limited access options in the future. From the patient perspective, there is a critical need for improved comprehensive education regarding kidney substitution and vascular access options and their potential complications. In order for large-scale implementation of the LP to occur, significant organizational change needs to occur, including resources for training clinicians on how to effectively communicate the goals of the LP with patients, processes to update, store, and access the LP, and means of sharing the LP across health systems and practices.

### Supplementary Information

Below is the link to the electronic supplementary material.

Supplementary file1 (DOCX 28 kb)
