# Supplementary material for: Barriers to Implementing the Kidney Disease Outcomes Quality Initiative End-Stage Kidney Disease Life Plan Guideline
Source: J Gen Intern Med. 2023 Jul 5;38(14):3198–208. doi: 10.1007/s11606-023-08290-5 (PMC10651571; doi:10.1007/s11606-023-08290-5)
Supplement: Supplementary file 1 — Supplementary file1 (DOCX 28 kb) [file 11606_2023_8290_MOESM1_ESM.docx]

**APPENDIX 1: PATIENT SEMI-STRUCTURED INTERVIEW GUIDE**

**1. Dialysis Experience**: Would you describe your experience so far with dialysis?

**2. Information Sources:** Where did you get information about your dialysis access options?

*Follow-up*: What would your ideal information source look like?

*Follow-up*: How closely, or not closely, have the information sources that you’ve encountered resembled your ideal information source?

*Follow-up*: If there were patients talking about their experience, would you want them to talk only about positive things, or negative things or both?

*Follow-up*: Do you want patients, or providers or families/care givers to talk?

*Follow-up*: What should be on a Frequently Asked Questions list?

**3. Experience with providers**: In general, as a dialysis patient, you interact with a lot of health care providers, such as nurses, doctors, aides and technicians. Could you describe any interactions or relationships with health care providers that stand out to you as being particularly positive?

*Follow-up*: Could you describe any interactions or relationships with health care providers that stand out to you as being particularly negative?

*Follow-up*: What would you change about the interactions that you have had with health care providers?

*Follow-up*: What would the ideal interaction with a physician look like?

**4. Life-Plan**: What have you heard about the ESKD Life-Plan?

*Follow-up*: What do you think about the Life-Plan?

**5. Shared-decision making preferences**: When it comes to making decisions about your dialysis vascular access, how much do you want to be involved in the decision making process?

*Follow-up*: How would you want to be involved with the multidisciplinary team of providers in making your Life-Plan?

*Follow*-*up*: Would you prefer to be directly involved with all the clinicians on the multidisciplinary team or would you prefer to interact with only one of the clinicians?

**6. Non-physician Providers**: Could you tell me about any non-physician providers that are important to your decision-making process about dialysis and vascular access?

*Follow-up*: How would you want those non-physician providers to be incorporated into the making of your Life-Plan?

**7. Social Support Structure**: Could you tell me about any other people in your life that help you to make decisions about dialysis and your vascular access?

*Follow-up*: How would you want those people to be involved in the development of your ESKD Life-Plan?

**8. Questions you would have asked**: What do you know now about vascular access that you wish you had known before?

*Follow-up:* Knowing what you know now, what questions would you have asked your doctor initially that you may not have asked before?

**9. Personal goals**: Thinking about your life with ESKD, what are the goals that you would like to achieve as a result of your treatment?

*Follow-up:* What aspects of your vascular access are most important to you?

*Follow-up*: What aspects of your vascular access have the biggest impact on you?

**10. Wrap-up**: Is there anything else about your experience with dialysis or your dialysis access or your thoughts about the ESKD Life-Plan that we have not covered today that you would like to talk about?

*Follow-up:* Is there anything that we have talked about that you want to discuss further?

*Follow-up*: Do you have any comments or questions about the interview?

**Baseline characteristics**. If baseline characteristics were not captured naturally during the course of the interview, specific characteristics will be collected at the conclusion of the interview:

- age
- sex
- race/ethnicity
- marital status
- education level
- employment
- co-morbidities
- years with kidney failure
- previous/current vascular accesses
- previous/current renal replacement modalities

**Appendix 2: CLINICIAN Semi-Structured Interview Guide**

1. **Provider and Practice Characteristics**: Would you describe your practice setting?

*Follow-up*: Would you describe your training background?

*Follow-up*: What kind of vascular access procedures and access-related procedures do you do?

2. **Management of Vascular Access**: Would you describe your general approach towards management of hemodialysis vascular access in ESKD patients?

*Follow-up*: How do you determine the ideal vascular access type for a given patient?

*Follow-up*: How do you decide if it is appropriate to do a revision procedure?

*Follow-up*: How do you decide if it’s time to give up on an access and attempt a new one?

3. **Multidisciplinary Teams**: What is your experience with working with multidisciplinary teams?

*Follow-up*: What do you think about multidisciplinary teams?

*Follow-up*: What do you think are the benefits of multidisciplinary teams?

*Follow-up*: What do you think are the drawbacks of multidisciplinary teams?

*Follow-up*: What do you think are the challenges of implementing multidisciplinary teams?

4. **Life-Plan**: How much do you know about the ESKD Life-Plan?

*Follow-up*: What are your impressions of the ESKD Life-Plan?

*Follow-up*: How easy or hard is it going to be to incorporate the ESKD Life-Plan into your practice?

*Follow-up*: What kind of structures do you have existing in your practice that will facilitate implementation of the ESKD Life-Plan?

*Follow-up*: What kind of structures do you think you would need to build into your practice in order to implement the ESKD Life-Plan?

5. **Life-Plan** **Multidisciplinary Providers**: What do you think about the specialty providers that are designated participants in the ESKD Life-Plan?

*Follow-up*: What other physician specialties do you think should be involved, if any?

*Follow-up*: What non-physician providers do you think should be involved, if any?

*Follow-up*: How do you feel about working with the other providers to make a unified plan for a patient?

*Follow-up*: How often do you disagree with another physician in your same specialty about how to manage a patient?

*Follow-up*: How often do you disagree with another physician in a different specialty about how to manage a patient?

*Follow-up*: When you have a disagreement with another physician about how to manage a patient, how do you resolve that?

*Follow-up*: How would you optimize communication among providers of the multidisciplinary team?

*Follow-up*: Who do you thing should chair the team?

6. **Patient Preferences**: What do you think about patient involvement in ESKD Life-Plan construction?

*Follow-up*: What do you think are ideal ways to incorporate patient preference into the Life-Plan?

*Follow-up*: Logistically speaking, what is the best way for the patient to interact with the multidisciplinary team?

*Follow-up*: What do you think about incorporating members of the patient’s social support structure into the Life-Plan construction?

*7*. **Wrap-up**: Is there anything about the ESKD Life-Plan or vascular access management that we haven’t discussed that you would like to bring up?

*Follow-up*: Is there anything that we have talked about that you want to discuss further?

*Follow-up*: Do you have any comments or questions about the interview?

**Baseline characteristics**. If baseline characteristics were not captured naturally during the course of the interview, specific characteristics will be collected at the conclusion of the interview:

- age
- sex
- race/ethnicity
- years in practice
- practice type

**APPENDIX 3. CONSOLIDATED CRITERIA FOR REPORTING QUALITATIVE STUDIES (COREQ) CHECKLIST**

| **Domain 1: Research team and reflexivity** | |
| --- | --- |
| Personal Characteristics | |
| 1. Interviewer/facilitator | KW and MK conducted the interviews and supervised the research |
| 1. Credentials | KW- MD, MS, PhD, MK- PhD, MPH |
| 1. Occupation | KW- vascular surgeon, professor of surgery, health services researcher  MK- health services researcher |
| 1. Gender | KW- female  MK- female |
| 1. Experience and training | KW- PhD trained in qualitative methodology  MK- PhD trained in qualitative methodology |
| Relationship with participants | |
| 1. Relationship established | Neither MK nor KW had any established relationship to a participant that they interviewed prior to study commencement |
| 1. Participant knowledge of the interviewer | The participants knew that MK and KW were the researchers leading this study. |
| 1. Interviewer characteristics | KW- research interest in vascular access. 50% of clinical practice is vascular access  MK- no clinical or personal experience with vascular access or renal failure |
| **Domain 2: study design** | |
| Theoretical framework | |
| 1. Methodological orientation and Theory | Grounded Theory |
| Participant Selection | |
| 1. Sampling | Purposive and snowball sampling were used. We aimed to purposefully sample patient participants of heterogeneous age, sex, race/ethnicity, marital status, education level, employment, co-morbidities, experience with hemodialysis, and duration of kidney failure. We snowball sampled by asking recruited patients to recommend other patients.  We aimed to purposefully sample clinicians from different specialties, practice settings, geographic diversity, gender, and years in practice. US clinicians were eligible if they were currently caring for patients with kidney disease. We aimed to recruit four pre-planned groups of individual clinicians specified by the LP (nephrologists, access surgeons, interventionalists, primary care providers) and included other clinicians (e.g., nurses and coordinators) as we pursued certain lines of inquiry. |
| 1. Method of approach | We recruited patients through the American Association of Kidney Patients, social media (e.g., Twitter), and the UCLA vascular surgery practice  We recruited clinicians through the investigators’ professional networks and social media |
| 1. Sample size | 21 clinicians  11 patients  2 care partners |
| 1. Non-participation | No people refused to participate |
| 1. Setting of data collection | Interviews were conducted using video teleconferencing (Zoom, Zoom Video Communications, San Jose, CA) or telephone |
| 1. Presence of non-participants | Nobody was present besides the participants and researchers that the researcher was aware of. As the interviews were not conducted in person, the researcher cannot confirm with 100% certainty that this is the case. |
| 1. Description of sample | Interviews were conducted between 4/2021 and 6/2022. Clinicians and patients were from all across the US with a range of age, race, specialty, experience with kidney disease. (See Table 1) |
| 1. Interview guide | The interview guides were purpose-developed by KW and MK. The interview guides were not pilot tested. They were adapted from previous similar interview guides. |
| 1. Repeat interviews | No repeat interviews were performed |
| 1. Audio/visual recording | For the interviews performed on teleconference, audio and visual was recorded. For the interviews performed over the phone, only audio was recorded. |
| 1. Field notes | Fields notes were made by the interviewer during and after the interview |
| 1. Duration | The interviews lasted between 40-60 minutes. |
| 1. Data saturation | Data saturation was determined by KW and MK to occur at 8 interviews in both the clinician and patient categories |
| 1. Transcripts returned | Transcripts were not returned to participants |
| **Domain 3: analysis and findings** | |
| Data analysis | |
| 1. Number of coders | 4 coders coded the data |
| 1. Description of coding tree | When 6 interviews were coded in each participant category, the investigators observed, through constant comparison, repeated concepts in the data. KW and MK independently grouped the initial codes into focused codes (constructs that succinctly capture important pattern in the data in relation to the research question and are more conceptual in nature.) The larger investigator group reached consensus on the final list of focused codes. We applied the focused codes to the remaining transcripts, with KW and MK checking to ensure consistency between coders.  We then mapped focused codes specific to LP implementation to relevant domains in the Consolidated Framework for Implementation Research (CFIR). |
| 1. Derivation of themes | Themes were derived according to CFIR |
| 1. Software | We used Dedoose v9.0.54 (SocioCultural Research Consultants, LLC, Manhattan Beach, CA) |
| 1. Participant checking | Participants did not provide feedback on the findings |
| Reporting | |
| 1. Quotations presented | Participant quotations were presented to illustrate themes and were identified by participant number. (See Tables 2-4) |
| 1. Data and findings consistent | There was consistency between the data presented and the findings |
| 1. Clarity of major themes | Major themes are clearly presented in the findings according to CFIR constructs |
| 1. Clarity of minor themes | Minor themes were not described or discussed |
